# Five Complete Genome Sequences Spanning the Dutch Streptococcus suis Serotype 2 and Serotype 9 Populations

**DOI:** 10.1128/MRA.01439-19

**Published:** 2020-02-06

**Authors:** Boas C. L. van der Putten, Thomas J. Roodsant, Martin A. Haagmans, Constance Schultsz, Kees C. H. van der Ark

**Affiliations:** a Amsterdam UMC, University of Amsterdam, Department of Global Health, Amsterdam Institute for Global Health and Development, Amsterdam, the Netherlands; b Amsterdam UMC, University of Amsterdam, Department of Medical Microbiology, Amsterdam, the Netherlands; c Amsterdam UMC, University of Amsterdam, Department of Clinical Genetics, Amsterdam, the Netherlands; University of Maryland School of Medicine

## Abstract

The zoonotic pathogen Streptococcus suis can cause septicemia and meningitis in humans. We report five complete genomes of Streptococcus suis serotype 2 and serotype 9, covering the complete phylogeny of serotype 9 Dutch porcine isolates and zoonotic isolates. The isolates include the model strain S10 and the Dutch emerging zoonotic lineage.

## ANNOUNCEMENT

Streptococcus suis is an opportunistic pathogen in pigs which can cause zoonotic infections. Human infections are predominantly caused by S. suis serotype 2 (1) and can lead to septicemia and meningitis (2). We recently identified a zoonotic S. suis serotype 2 clone belonging to clonal complex 20 (CC20), which emerged from a nonzoonotic serotype 9 CC16 clone (3) in the Netherlands. To facilitate further research on the zoonotic potential of S. suis, we sequenced the genomes of S. suis serotype 9 CC16 and CC20 strains, isolated from diseased pigs, and three serotype 2 strains, including strain S10 (CC1, pig) and two CC20 strains, one each from human and porcine infections (Table 1). Data were generated using Illumina and Nanopore MinION sequencing technologies.

**TABLE 1:** Isolate details, genome information, and accession numbers

Isolate	Isolation source	Serotype	Clonal complex	Genome length (bp)	GC content (%)	No. of total CDSs^a^	Nanopore read *N*_50_ (bp)	No. of Nanopore reads	Nanopore coverage (×)	Nanopore run accession no.	Illumina run accession no.	Assembly accession no.
861160	Human CSF^b^	2	20	2,148,824	41.10	2,029	13,589	7,557	23	ERR3664732	ERR1055554	GCA_902702745
GD-0001	Diseased pig	2	20	2,125,468	41.24	2,014	25,831	10,490	54	ERR3664733	ERR1055586	GCA_902702785
9401240	Diseased pig	9	20	2,195,215	41.43	2,036	11,192	30,418	60	ERR3664735	ERR1055578	GCA_902702775
GD-0088	Diseased pig	9	16	2,298,012	41.20	2,213	7,657	15,321	27	ERR3664734	ERR1055627	GCA_902702765
S10	Diseased pig	2	1	2,048,275	41.32	1,952	15,208	20,251	72	ERR3664731	ERR1055646	GCA_902702755

a CDSs, coding sequences.

b CSF, cerebrospinal fluid.

S. suis was grown overnight in Todd-Hewitt broth supplemented with yeast extract (THY), and genomic DNA was isolated using the Qiagen MagAttract high-molecular-weight (HMW) DNA extraction kit. The sequence library was constructed using the native barcoding (catalog number EXP-NBD114) and ligation sequencing (catalog number SQK-LSK109) kits (Oxford Nanopore). DNA was repaired and A tailed using NEBNext formalin-fixed, paraffin-embedded (FFPE) DNA repair mix and the NEBNext Ultra II end repair/dA-tailing module (New England BioLabs). A barcode was ligated to the A-tailed DNA using blunt/TA ligase master mix (New England Biolabs). Sequence adapters were ligated to barcoded samples pooled by equal mass with Quick T4 DNA ligase (New England BioLabs). The library was loaded on the flow cell (FLO-MIN106D [R9]) and sequenced using MinKNOW fast base calling version 3.5.5. Default parameters were used for all tools except where noted otherwise. Illumina data were available from our previous study (Table 1) (3).

Illumina read filtering was performed using fastp version 0.20.0 (4). MinION reads were filtered for quality and length using Filtlong version 0.2.0 (5), using the filtered Illumina reads as reference. FastQC version 0.11.8 was used for quality control (6). Illumina and MinION reads were used in a hybrid assembly using Unicycler version 0.4.8, which also performs assembly trimming, circularizing, and rotating (7). Assembly statistics were collected using Quast version 4.6.3 (8). Coverage was assessed using Minimap2 version 2.17 (9), SAMtools version 1.9 (10), and BEDTools version 2.29.0 (11). The complete genomes were annotated using Prokka version 1.14.0 (12). Multilocus sequence typing (MLST) was performed using mlst version 2.17.6 (13). For workflow management, Snakemake version 5.7.1 (14) was used. The pipeline is freely available from https://github.com/boasvdp/MRA_Streptococcus_suis.

Genomes of all five strains consisted of a single chromosome ranging from 2,042,889 to 2,292,626 bp with a GC content of 41.10 to 41.43% and a coverage of 23 to 72×, determined using Nanopore data (Table 1).

Draft assemblies of the five strains were 46 to 74 kbp smaller than the complete genomes. Mapping the draft genomes to the complete genomes revealed no missing regions in the draft genomes. The draft genomes are likely smaller than the complete genomes due to the collapse of repeats, which has been described before (15).

### Data availability.

Nanopore, fastq, and fast5 data, as well as the assembled genome sequences, have been deposited in ENA under the accession numbers listed in Table 1 and study number PRJEB35407.
