# Correction for van der Putten et al., “Five Complete Genome Sequences Spanning the Dutch Streptococcus suis Serotype 2 and Serotype 9 Populations”

**DOI:** 10.1128/mra.00951-22

**Published:** 2022-10-27

**Authors:** Boas C. L. van der Putten, Thomas J. Roodsant, Martin A. Haagmans, Constance Schultsz, Kees C. H. van der Ark

## AUTHOR'S CORRECTION

Volume 9, no. 6, e01439-19, 2020, https://doi.org/10.1128/MRA.01439-19.

Page 1, line 7: “CC20” should read “CC220.”

Page 2, Table 1, line 3, column 4: “20” should read “220.”

Page 2, Table 1, line 5, column 2: “Diseased pig” should read “Healthy pig.”

